# Study of the efficacy of antimalarial drugs delivered inside targeted immunoliposomal nanovectors

**DOI:** 10.1186/1556-276X-6-620

**Published:** 2011-12-07

**Authors:** Patricia Urbán, Joan Estelrich, Alberto Adeva, Alfred Cortés, Xavier Fernàndez-Busquets

**Affiliations:** 1 Nanobioengineering Group, Institute for Bioengineering of Catalonia, Baldiri Reixac 10-12, Barcelona, E08028, Spain; 2 Nanoscience and Nanotechnology Institute (IN2UB), University of Barcelona (UB), Martí i Franquès 1, Barcelona, E08028, Spain; 3 Barcelona Centre for International Health Research (CRESIB), Hospital Clínic-Universitat de Barcelona, Rosselló 132, Barcelona, E08036, Spain; 4 Departament de Fisicoquímica, Facultat de Farmàcia, University of Barcelona, Av. Joan XXIII, s/n, Barcelona, E08028, Spain; 5 Scientific and Technological Centres, University of Barcelona, Baldiri Reixac 10-12, Barcelona, E08028, Spain; 6 Institute for Research in Biomedicine, Barcelona Science Park, Baldiri Reixac 10-12, Barcelona, E08028, Spain; 7 Institució Catalana de Recerca i Estudis Avançats (ICREA), Passeig Lluís Companys 23, Barcelona, E08018, Spain

**Keywords:** antimalarial chemotherapy, chloroquine, fosmidomycin, half-antibodies, immunoliposomes, malaria, nanomedicine, targeted drug delivery

## Abstract

Paul Ehrlich's dream of a 'magic bullet' that would specifically destroy invading microbes is now a major aspect of clinical medicine. However, a century later, the implementation of this medical holy grail continues being a challenge in three main fronts: identifying the right molecular or cellular targets for a particular disease, having a drug that is effective against it, and finding a strategy for the efficient delivery of sufficient amounts of the drug in an active state exclusively to the selected targets. In a previous work, we engineered an immunoliposomal nanovector for the targeted delivery of its contents exclusively to *Plasmodium falciparum*-infected red blood cells [pRBCs]. In preliminary assays, the antimalarial drug chloroquine showed improved efficacy when delivered inside immunoliposomes targeted with the pRBC-specific monoclonal antibody BM1234. Because difficulties in determining the exact concentration of the drug due to its low amounts prevented an accurate estimation of the nanovector performance, here, we have developed an HPLC-based method for the precise determination of the concentrations in the liposomal preparations of chloroquine and of a second antimalarial drug, fosmidomycin. The results obtained indicate that immunoliposome encapsulation of chloroquine and fosmidomycin improves by tenfold the efficacy of antimalarial drugs. The targeting antibody used binds preferentially to pRBCs containing late maturation stages of the parasite. In accordance with this observation, the best performing immunoliposomes are those added to *Plasmodium *cultures having a larger number of late form-containing pRBCs. An average of five antibody molecules per liposome significantly improves in cell cultures the performance of immunoliposomes over non-functionalized liposomes as drug delivery vessels. Increasing the number of antibodies on the liposome surface correspondingly increases performance, with a reduction of 50% parasitemia achieved with immunoliposomes encapsulating 4 nM chloroquine and bearing an estimated 250 BM1234 units. The nanovector prototype described here can be a valuable platform amenable to modification and improvement with the objective of designing a nanostructure adequate to enter the preclinical pipeline as a new antimalarial therapy.

## Introduction

**Figure 1:**
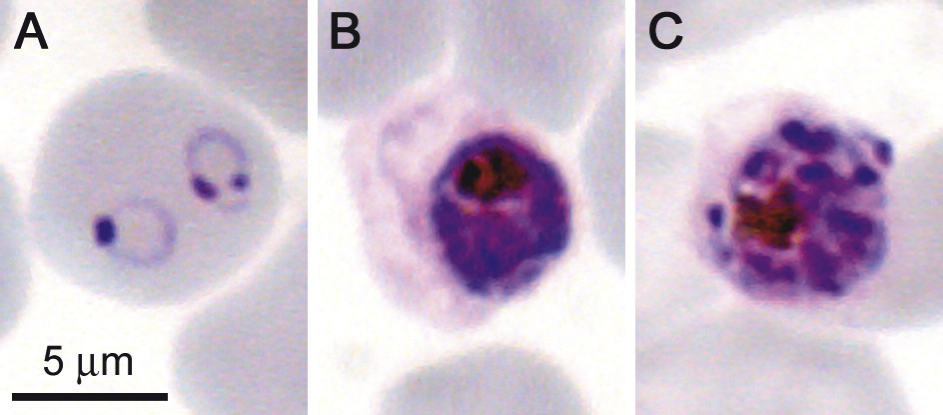
**Giemsa staining of *Plasmodium*-infected RBCs**. At the ring (**A**), trophozoite (**B**), and schizont (**C**) stages of *P. falciparum*.

Malaria is an acute and/or chronic infection caused by protozoans of the genus *Plasmodium*. Clinical manifestations are fever, chills, prostration, and anemia, whereas severe disease can include metabolic acidosis, cerebral malaria, and multiorgan system failure, and coma and death may ensue. More than 40% of the world's population lives with some risk of contracting malaria, with most recent estimates suggesting several hundred million clinical cases and 800,000 deaths each year [1,2], of which the large majority are children below 5 years [3,4]. The recent call for the elimination and eradication of the disease requires research from multiple fronts, including developing strategies for the efficient delivery of new medicines [5]. Four species cause diseases in humans: *P. vivax*, *P. ovale*, *P. malariae*, and *P. falciparum*, with the latter causing the most deadly and severe cases. In the life cycle of *Plasmodium *parasites (for a review, see Tuteja [6]), the female *Anopheles *mosquito inoculates, during a bite, *Plasmodium *sporozoites that bind to and infect hepatocytes and proliferate into thousands of merozoites in the liver. Merozoites rupture from the hepatocytes and invade red blood cells [RBCs], where they develop first into rings and then into the late forms, trophozoites and schizonts (Figure 1). Schizont-infected RBCs burst and release more merozoites, which start the blood cycle again. Because the blood-stage infection is responsible for all symptoms and pathologies of malaria, *Plasmodium*-infected RBCs [pRBCs] are a main chemotherapeutic target [7].

The need to develop new strategies to treat malaria is urgent if one considers that the cases of resistance to current antimalarial agents increase, especially in zones in which *P. falciparum *is endemic, and calls for combined therapy approaches [5,8]. Several drugs show different degrees of toxicity, which limits their use because current administration forms release the free compound in the blood and offer little specificity regarding the targeted cells [9]. Consequently, to achieve therapeutic levels that extend over time, the initial concentration of the drug in the body should be high. On the other hand, if the administered chemical has unspecific toxicity, the low doses required contribute to the development of resistant parasite strains [10]. The challenge of drug delivery is the liberation of adequate doses of therapeutic agents to a specific target site at the right time in a safe and reproducible manner [11]. A number of mechanisms can provide controlled release, including transdermal patches, implants, inhalation systems, bioadhesive systems, and nanoencapsulation [12-14], but few of these have been designed for specificity. Taking into account the peculiarities of pRBCs, lipid-based nanocarriers have been one of the most promising approaches for the targeted delivery of antimalarial drugs [15].

Liposomes are synthetic lipid bilayer-enclosed structures up to several hundred nanometers in diameter that can improve the delivery of bioactive molecules by functioning as circulating microreservoirs for sustained release [16,17]. Liposomes bearing cell-specific recognition ligands on their surfaces have been widely considered as drug carriers in therapy [18,19], and liposome encapsulation has been assayed for the targeted delivery of compounds against murine malaria [20-22]. Liposomal nanovessels incorporating a *P. berghei *amino acid sequence have been shown to greatly increase their targeting to the liver [23,24], suggesting that they can be an adequate vector to channel antimalarials towards the hepatocyte stages of the parasite.

In a previous work [25], we engineered an immunoliposomal nanovector for the targeted delivery of its contents exclusively to pRBCs. Two hundred-nanometer liposomes loaded with quantum dots were covalently functionalized with oriented, specific half-antibodies against *P. falciparum *late form-infected pRBCs. In less than 90 min, 100% of late form-containing pRBCs and 0% of noninfected RBCs in living *P. falciparum *cultures were infiltrated by the content of targeted immunoliposomes. Here, we present a quantitative study of the efficacy of this nanovector in ameliorating the activity of the antimalarial drugs chloroquine and fosmidomycin, and discuss its characteristics and room for improvement regarding future animal assays that might place this prototype on the threshold of clinical trials.

## Materials and methods

### Materials

Except where otherwise indicated, all reagents were purchased from Sigma-Aldrich Corporation (St. Louis, MO, USA). The commercial monoclonal antibody BM1234 against *P. falciparum *pRBCs was obtained from Acris Antibodies GmbH (Herford, Germany).

### Liposome and immunoliposome preparation

Liposomes were prepared by the lipid film hydration method [26], with the lipid formulation 1,2-dioleoyl-*sn*-glycero-3-phosphatidylcholine [DOPC] (Avanti Polar Lipids, Inc., Alabaster, AL, USA)/cholesterol 80:20, as described previously [25]. The dry lipids were hydrated at 37°C in phosphate-buffered saline [PBS] containing the antimalarial drug to obtain a concentration of 10 mM lipid. For the coupling of targeting antibodies to liposomes, we followed established protocols [27] that use 1,2-dipalmitoyl-*sn*-glycero-3-phosphoethanolamine-*N*-[4-(*p*-maleimidophenyl)butyramide] [MPB-PE] (Avanti Polar Lipids, Inc.) to incorporate proteins into liposomes (DOPC/cholesterol/MPB-PE 77.5:20:2.5, 10 mM lipid) through the reaction of the maleimide group in the lipid with a thiol group from the ligand [25].

### Quantification of chloroquine and fosmidomycin

The amount of chloroquine and fosmidomycin encapsulated inside liposomes was determined using high-performance liquid chromatography with tandem mass spectrometry [HPLC-MS/MS]. Prior to analysis, lipids were extracted following stardard protocols [28] in order to release the entrapped drug. Briefly, 100 μl of liposome sample was mixed with 225 μl of cold methanol and 125 μl of chloroform. Phase separation was achieved by adding 125 μl of 0.1 M HCl and 125 μl of chloroform. After mixing thoroughly, the samples were spun down at 7,500 × *g *for 5 min. The upper water-methanol layer, containing the drug that had been encapsulated inside liposomes, was collected and stored at -20°C until analysis. HPLC analysis was performed in an Alliance 2695 chromatographic system (Waters Corporation, Milford, MA, USA) using an Atlantis dC18 analytical column (internal diameter [i.d.] 150 × 2.1 mm, 5 μm, Waters Corporation) for chloroquine and a Luna C18 analytical column (i.d. 150 × 2.0 mm, 5 μm, Phenomenex, Torrance, CA, USA) for fosmidomycin, with mobile phase A (0.2% formic acid in water) and B (acetonitrile). The linear gradient for the determination of chloroquine at a flow rate of 0.3 ml/min was (% mobile phase B/min) 0/0, 10/2, 40/17, and 90/22 whereas for the determination of fosmidomycin at a flow rate of 0.4 ml/min, it was 0/0, 0/5, and 95/10. The HPLC system was coupled to a SCIEX API 365 Triple Quadrupole mass spectrometer equipped with a Turbo Ion Spray ion source (PerkinElmer, Waltham, MA, USA). For quantification purposes, data were collected in the multiple reaction monitoring mode with positive-ion detection for chloroquine and negative-ion detection for fosmidomycin, tracking the transition of the parent and product ions specific for each compound: 320.3/141.9 and 320.3/246.7 for chloroquine and 182.0/79.0 and 182.0/136.0 for fosmidomycin.

### Cryo-electron microscopy

For cryo-electron microscopy analysis of the preparations of liposomes, a thin aqueous film was formed by dipping a glow-discharged holey carbon grid in the liposome suspension and then blotting the grid against a filter paper. The resulting thin sample films spanning the grid holes were vitrified by plunging the grid (kept at 100% humidity and room temperature) into ethane, which was maintained at its melting point with liquid nitrogen, using a Vitrobot (FEI Company, Eindhoven, The Netherlands). The vitreous films were transferred to a Tecnai F20 transmission electron microscope [TEM] (FEI Company) using a cryotransfer (Gatan, Inc., Pleasanton, CA, USA), and the samples were observed in a low-dose mode. Images were acquired at 200 kV at a temperature between -170°C and -175°C, using low-dose imaging conditions not exceeding 20 *e*^-^/Å^2^, with a 4,096 × 4,096 pixel CCD Eagle camera (FEI Company), and digitized with the Tecnai Image Acquisition program.

### *Plasmodium falciparum *cell culture and growth inhibition assays

The *P. falciparum *3D7 strain was grown *in vitro *in group B washed human RBCs prepared as described elsewhere [25] using previously described conditions [29]. Briefly, parasites (thawed from glycerol stocks) were cultured at 37°C in Petri dishes containing RBCs in RPMI complete medium under a gas mixture of 92% N_2_, 5% CO_2_, and 3% O_2_. Synchronized cultures were obtained by 5% sorbitol lysis [30], and the medium was changed every 2 days maintaining 3% hematocrit. For culture maintenance, parasitemias were kept below 5% late forms by dilution with washed RBCs. For standard growth inhibition assays, parasitemia was adjusted to 1.5% with more than 90% of parasites at the ring stage after sorbitol synchronization. For modified growth inhibition assays, synchronized cultures were incubated for 24 h before addition of the drug to allow for the appearance of late forms presenting the epitope recognized by BM1234 bound to immunoliposomes. Two hundred microliters of these living *Plasmodium *cultures were plated in 96-well plates and incubated for 48 h at 37°C in the presence of free drugs and drugs encapsulated in liposomes and immunoliposomes at a final concentration of 100 μM lipid. Parasitemia was determined by microscopic counting of blood smears or by fluorescence-assisted cell sorting [FACS]. Smears were fixed in methanol for a few seconds and then stained for 10 min with Giemsa (Merck Chemicals, Darmstadt, Germany) diluted 1:10 in Sorenson's buffer, pH 7.2. After washing with distilled water and drying, the ratio of the infected vs. noninfected RBCs was determined by microscopic analysis. For FACS analysis, noninfected RBCs and samples containing pRBCs were diluted to a final concentration of 1 to 10 × 10^6 ^cells/ml. The cell suspension was stained with SYTO 11 (0.5 mM stock in DMSO, Molecular Probes, Eugene, OR, USA) to a final concentration of 0.5 μM, and samples were incubated for 5 to 10 min prior to analysis in a Cytomics FC 500 MPL (Beckman Coulter, Inc., Fullerton, CA, USA) set up with the standard configuration. Excitation of the sample was done using a 488-nm, air-cooled, argon-ion laser at 15-mW power using forward and side scatter to gate the RBC population, and SYTO 11 green fluorescence (525 nm) was collected in a logarithmic scale. The single-cell population was selected on a forward-side scattergram, and the green fluorescence from this population was analyzed. Parasitemia was expressed as the number of parasitized cells per 100 erythrocytes.

### Statistical analysis

Data are presented as the mean ± standard error of at least three independent experiments, and the corresponding standard errors in histograms are represented by error bars. Statistical analyses were performed using the Statgraphics Centurion XVI.I data analysis and statistical software. The parametric Student's *t *test was used to compare two independent groups when data followed a Gaussian distribution. Otherwise, the nonparametric Mann-Whitney test was used. Differences were considered significant when the *p *value was ≤ 0.05.

### Confocal fluorescence microscopy

For immunofluorescence assays, pRBC smears were fixed in ice for 2 min in acetone/methanol (90:10) and incubated with 2 μg BM1234 monoclonal antibody/ml PBS containing 0.75% *w*/*v *BSA for 90 min at 37°C. After PBS washing steps, primary antibodies were detected with a secondary fluorescent antibody Alexa Fluor 488 goat anti-mouse F(ab')_2 _(Molecular Probes). Parasite nuclei were stained with 4'6-diamino-2-phenylindole [DAPI] (Invitrogen Corporation, Carlsbad, CA, USA) during the secondary antibody incubation, and the RBC membrane was labeled with a wheat germ agglutinin [WGA]-tetramethylrhodamine conjugate (Molecular Probes). After the corresponding incubations and PBS washing steps, the samples were finally mounted with Mowiol (Calbiochem, Merck Chemicals) following standard protocols [31]. The samples were imaged with a TCS SP5 laser scanning confocal microscope equipped with an acoustic optical beam splitter (Leica Microsytstems Inc., Buffalo Grove, IL, USA), a DMI6000 inverted microscope (Leica Microsytstems Inc.), blue diode (405 nm), Ar (458/476/488/496/514 nm), diode-pumped solid-state (561 nm), and HeNe (594/633 nm) lasers, and APO ×63 oil (NA 1.4) or glycerol (NA 1.3) immersion objective lenses. DAPI, Alexa Fluor 488, reflection (for hemozoin detection), and WGA-rhodamine images were acquired sequentially using 405, 488, 488, and 561 laser lines and emission detection ranges 415 to 480 nm, 500 to 550 nm, 480 to 500 nm, and 571 to 625 nm, respectively, with the confocal pinhole set at 1 airy unit. Bright field transmitted light images were acquired simultaneously at 400 Hz in a 512 × 512 pixel format, ×8 zoom, and a pixel size of 60 × 60 nm.

## Results and discussion

**Figure 2:**
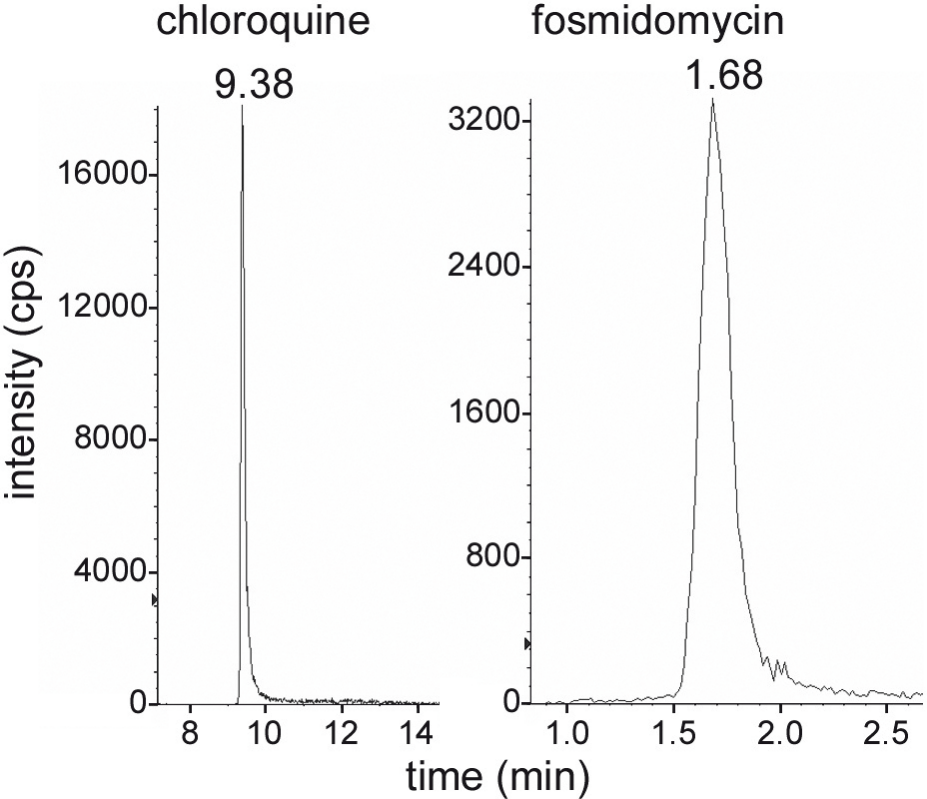
**HPLC chromatograms showing the retention times of chloroquine and fosmidomycin extracted from immunoliposomes**.

In preliminary *in vitro *assays, chloroquine showed an improved efficacy as an antimalarial when delivered inside targeted immunoliposomes [25]. However, difficulties in determining the exact concentration of the drug due to the low administered amounts prevented an accurate estimation of the nanovector performance. Here, we have established an HPLC method for the reliable quantification of chloroquine and fosmidomycin with good linearity ranges of 0.1 to 100 ng/ml (*Y *= 0.0223X - 1.98 × 10^-13^, *r*^2 ^= 0.9942) and 5 to 2,500 ng/ml (*Y *= 246X, *r*^2 ^= 0.9997), respectively (Figure 2). The detection limits obtained have been 0.04 ppb for chloroquine and 1 ppb for fosmidomycin.

**Figure 3:**
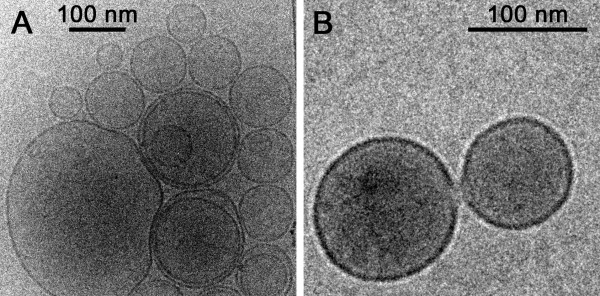
**Cryo-TEM images of liposomes**.

For immunoliposome assembly, different lipid combinations had been tested in order to establish a formulation with low hemolytic activity and low general cytotoxicity, which is DOPC/cholesterol/MPB-PE 77.5:20:2.5 [25]. Liposomes formed in this way were stable and generally did not coalesce even after high-speed centrifugation (Figure 3A), but occasionally, fusion events were observed (Figure 3B). Although dynamic light scattering analysis indicated that the liposome population had a mean diameter of 200 nm and a lower limit of 100 nm, TEM images showed a significant number of smaller liposomes down to 50 nm across. Most liposomes were unilamellar but a substantial fraction of them (about 10%) were enclosed by two or more lipid bilayers.

**Figure 4:**
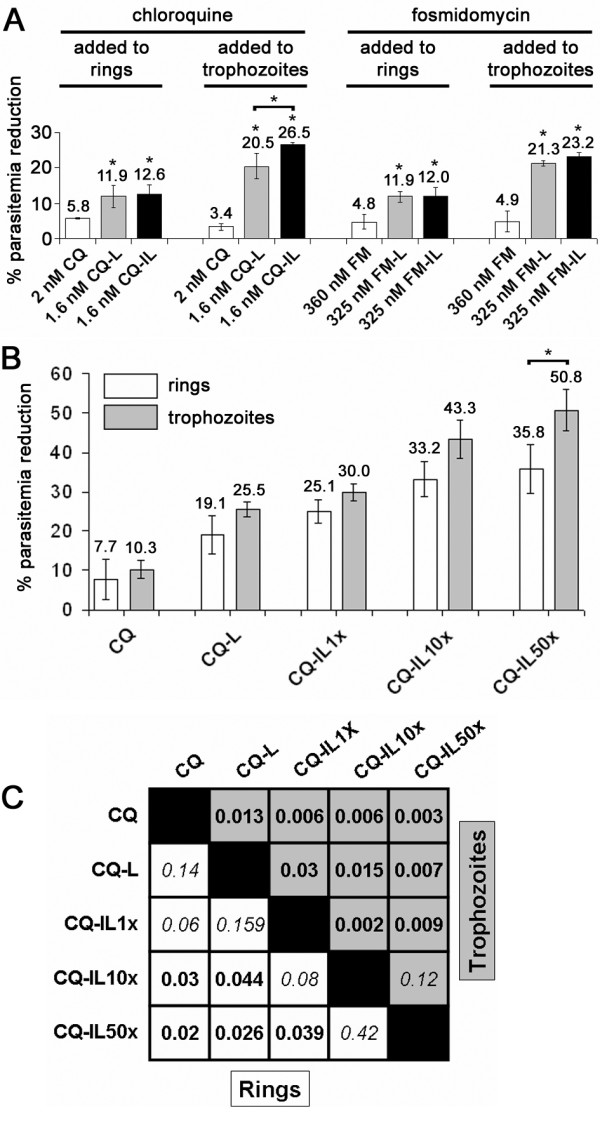
**Growth inhibition assays**. (**A**) Effect on *P. falciparum *viability of chloroquine (CQ) and fosmidomycin (FM), free or encapsulated in liposomes (L) or immunoliposomes (IL) and added at the ring or the trophozoite stage. The values express percentage of reduction respective to the parasitemia of controls without drug added. Asterisks on top of individual bars indicate significant differences (*p *≤ 0.05) in relation to the corresponding control sample of the non-encapsulated drug. (**B**) Effect on *P. falciparum *viability of increasing amounts of BM1234 antibody on chloroquine-containing immunoliposomes. The amount of chloroquine added to the culture was 4 nM in all samples. 1×, 10×, and 50 × correspond respectively to 5, 50, and 250 estimated antibody molecules per liposome. The asterisk indicates a significant difference (*p *≤ 0.05). (**C**) Grid showing the *p *values within the trophozoite and ring sample groups in the experiment from panel B. Bold numbers indicate a significant difference (*p *≤ 0.05).

The targeting antibody BM1234 used in this work is specific for pRBCs infected by the trophozoite and schizont forms, but it does not bind significantly to ring-stage pRBCs [25]. For this reason, we speculated that a BM1234-mediated targeted delivery of immunoliposome-encapsulated drugs would be more efficient if administered to trophozoites rather than to rings. In a classical *Plasmodium *growth inhibition assay, the drug is added at the ring stage, and after 48 h of incubation throughout a complete erythrocytic cycle, parasitemia is determined. Here, we have also assayed the addition of our targeted nanovectors at the trophozoite stage (24 h later than in the standard protocol), determining parasitemia by FACS analysis and Giemsa-stained blood smears after a further 48 h of incubation. The results obtained (Figure 4A) show that in growth inhibition assays performed 48 h after being administerd to pRBC cultures, 2 nM chloroquine or 360 nM fosmidomycin in solution reduces parasitemia by 3% to 6% when added at either the ring or the trophozoite stage. Liposomal chloroquine and fosmidomycin used at respective final concentrations of 1.6 and 325 nM reduce parasitemia by > 10% when added at the ring stage and by > 20% when added at the trophozoite stage. The best results have been obtained with chloroquine and fosmidomycin encapsulated in immunoliposomes, which resulted in *ca*. eightfold and fivefold improvement for encapsulated drug concentrations 80% and 90% that of the free compound, respectively. On an average, encapsulation in immunoliposomes as described here improves drug efficacy by an order of magnitude: similar drug amounts kill 10 times more parasites if encapsulated, and 10 times less encapsulated drug eliminates approximately the same number of parasites than the free compound: when added at the trophozoite stage, 20 nM soluble chloroquine kills 28.1 ± 0.6% of parasites vs. 26.5 ± 0.5% for the immunoliposome-encapsulated drug at 1.6 nM final concentration. The efficacy of the nanovector is particularly relevant in the case of chloroquine, which has an endogenous carrier across human erythrocyte membranes that accumulates the drug selectively in these cells [32]. As expected, because the targeting antibody used here does not recognize ring-infected pRBCs, the efficacy of the immunoliposomes is much better when administered at the trophozoite stage. This observation has clinical implications related with the synchrony of falciparum malaria infection in humans, namely the administration of such stage-specific nanovectors should be timed at the moment when the targeted form is at its highest concentration in the blood. Although this can be readily determined by FACS analysis, Giemsa staining and a microscopic observation provide a more affordable way to quickly estimate the intraerythrocytic phase of the infection.

We have calculated that the protocol used for the preparation of the samples from Figure 4A results in an average number of five antibody molecules per liposome [25], and the data presented here indicate that this is the minimal antibody/liposome ratio that significantly improves the activity of immunoliposomal drugs. This ratio was sufficient to provide a complete pRBC targeting specificity according to the delivery of immunoliposome-contained quantum dots [25], but the same amount of targeting antibody in immunoliposomes does not result in a 100% elimination of parasitemia at the low antimalarial drug concentrations assayed. Because the role of targeting antibodies is most likely to anchor liposomes to pRBCs when a random collision occurs, the *in vitro *efficacy of immunoliposomes as drug delivery vessels can likely be increased by incorporating more antibodies on their surfaces. As shown in Figure 4B, C, increasing the antibody concentration in immunoliposomes containing 4 nM chloroquine results in a corresponding significant increase of nanovector performance, reaching a clearance of 50% parasitemia for liposomes studded with an estimated 250 antibody molecules. However, keeping the antibody concentration as low as possible will contribute to reduce the immune response towards plasma-circulating immunoliposomes. Thus, when designing *in vivo *assays, a balance has to be reached between a sufficiently high density of targeting antibody for efficient drug delivery and levels below those triggering fast lymphocyte uptake. Since another factor affecting the number of random encounters between liposomes and pRBCs is the movement of both particles in solution, we expect that the turbulent mixing in the bloodstream will permit the use of low targeting antibody amounts. As mentioned above, BM1234 immunoliposomes deliver their contents to all pRBCs present in a sample, but when loaded with drug, they are not able to eliminate all *Plasmodium *parasites. It is possible that an increased performance can be achieved even with a few targeting antibody molecules per liposome if the amount of the encapsulated drug could be increased. In this regard, Ashley et al. [33] have assembled liposome-enclosed silica nanoparticles termed protocells, able to carry high anticancer drug payloads, with a single protocell having the capacity to kill a cancer cell. It would be worth exploring if such protocells can also be used as antimalarial drug-containing structures.

**Figure 5:**
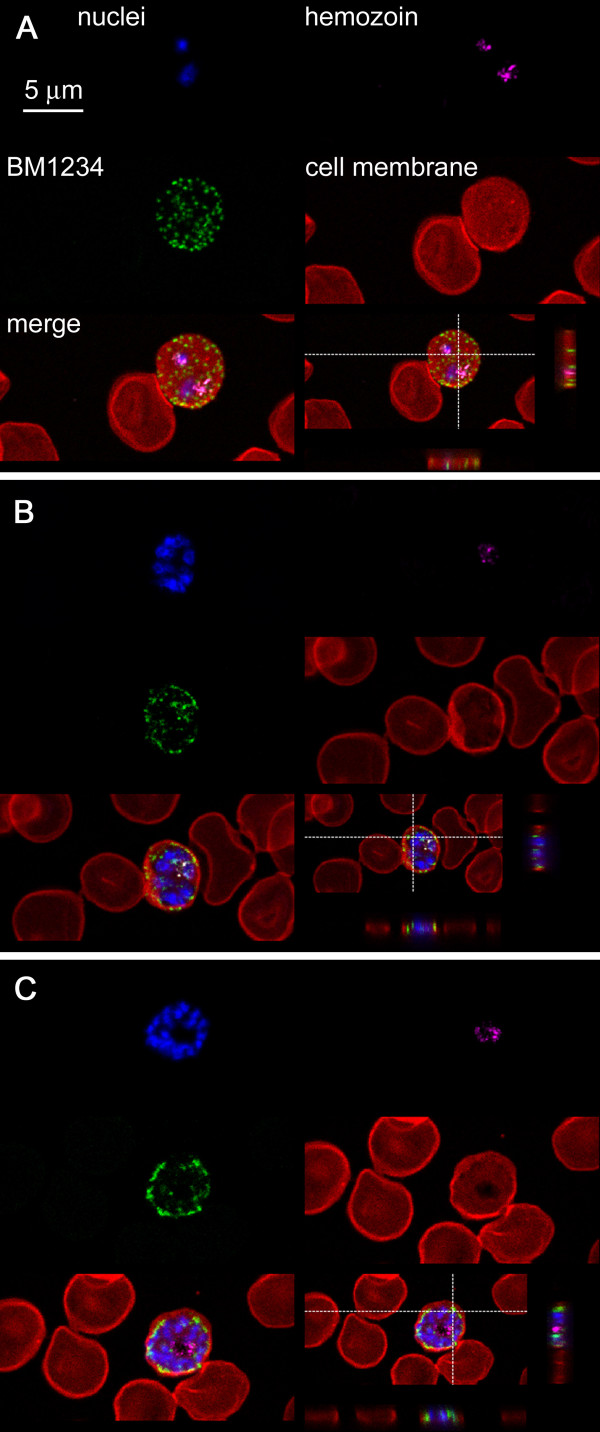
**Confocal fluorescence microscopy study of the subcellular localization of BM1234**. Early trophozoites (**A**), late trophozoites (**B**), and schizonts (**C**). Monoclonal antibody BM1234 was added to acetone/methanol-fixed *P. falciparum *cultures of the 3D7 strain, and its binding was detected by confocal fluorescence microscopy using a secondary antibody (green). DAPI (nuclei, in blue) and hemozoin fluorescence (pink) are used to indicate pRBCs. The RBC plasma membrane is shown in red. The four upper panels correspond to a single confocal section and are overlaid in the lower left panel. The lower right panel shows two perpendicular cross sections throughout the stack of images.

The preferential binding of BM1234 to trophozoites and schizonts suggested a differential subcellular localization of the corresponding antigen throughout the intraerythrocytic cycle of *P. falciparum*. This antibody had been selected for its binding to external features of pRBCs [25], which was a necessary condition for the targeting of these cells *in vivo*. When pRBCs are fixed with acetone/methanol prior to immunocytochemistry, BM1234 is observed to bind internal pRBC structures also (Figure 5). These are likely corresponding to regions in the endomembrane system built by *Plasmodium *that delineate the trafficking of the BM1234 antigen from its synthesis towards its final location in the pRBC plasma membrane. Alternatively, they could also represent internalization routes of externally exposed antigens. As expected, the subcellular localization of BM1234 antigens is changing during the intraerythrocytic cycle. A dominant spotted pattern in early trophozoites (Figure 5A) is consistent with the binding to pRBC intracellular membranous structures termed Maurer's clefts. In late trophozoites (Figure 5B) and especially in schizonts (Figure 5C), BM1234 binds structures on the periphery of the pRBC.

The remarkable capacity of liposomes to inject their contents into pRBCs presumably has its basis on alterations of the pRBC plasma membrane, rendering it less elastic and thus limiting the rebounding of colliding liposomes. The resulting slightly longer interactions, a phenomenon exacerbated if targeting antibodies are present, likely allow enough time for the physical phenomenon of lipid bilayer fusion to occur. This hypothesis could also explain why liposomes devoid of the antibody do perform better when added at the trophozoite stage since the membrane of ring-containing pRBCs is more similar to uninfected erythrocytes than that of pRBCs infected by later forms.

## Conclusions

Next in our agenda is to advance towards a nanovector-based antimalarial delivery strategy suitable to enter preclinical trials. Liposomal nanovectors are adequate for a parenteral delivery, indicated in cases of complicated malaria, those at risk of developing severe disease, or if the patient is vomiting and unable to take oral antimalarials. Parenteral treatment can also be required in the *last mile *of a malaria eradication protocol for the single-dose, individualized administration of highly toxic drugs specifically targeted to pRBCs with good accuracy with the objective of eliminating remaining multiresistant strains. The nanovector prototype described here can improve *ca*. tenfold the activity of hydrosoluble antimalarial drugs, and it is amenable to improvement or adaptation through modification of some of its parts, e.g., better antibodies or targeting molecules, different nanocapsule structures, or new antimalarial drugs. Finally, BM1234 immunoliposomes can also be an efficient drug delivery vessel for the administration of liposoluble drugs against *P. falciparum*; unlike chloroquine and fosmidomycin, the solubility of hydrophobic compounds will be low when free in plasma, but can be significantly increased when incorporated in the lipid bilayers of liposomes.

## Competing interests

The authors declare that they have no competing interests.

## Authors' contributions

PU carried out the inhibition assays, performed the microscopy analysis, participated in the design of the study and data analysis, and drafted the manuscript. JE participated in the liposome design and assembly and in the data analysis. AA carried out the chloroquine and fosmidomycin determination. AC participated in the *P. falciparum *culture preparation and in the analysis of subcellular localizations. XF-B conceived and coordinated the study, participated in the design of the study and data analysis, and drafted the manuscript. All authors read and approved the final manuscript.
